# Relapsing Salmonella Meningitis in an Infant: A Case Report and Review of the Literature

**DOI:** 10.1155/crpe/6662180

**Published:** 2026-08-17

**Authors:** Sonia Alexiadou, Elpis Mantadakis, Ioanna Irakleidou, Stavroula Kalatzi, Eleni Myrtzaki, Evlampia A. Psatha, Dimitrios Cassimos

**Affiliations:** ^1^ Department of Pediatrics, University General Hospital of Alexandroupolis, Alexandroupolis, Thrace, Greece; ^2^ Department of Medicine, School of Health Sciences, Democritus University of Thrace Faculty of Medicine, Alexandroupolis, Thrace, Greece, duhs.edu.pk; ^3^ Department of Medicine, School of Health Sciences, Democritus University of Thrace, Alexandroupolis, Thrace, Greece, duth.gr; ^4^ Department of Radiology, University General Hospital of Alexandroupolis, Alexandroupolis, Thrace, Greece

## Abstract

Accurate diagnosis of bacterial meningitis is critical, particularly in young febrile infants. Although commercially available multiplex PCR assays enable rapid pathogen detection, these typically do not include *Salmonella* spp. or other Enterobacteriaceae, which may result in missed diagnoses and/or inappropriate treatment duration. We report the case of a 2‐month‐old female infant with *Salmonella enteritidis* meningitis and concurrent bacteremia. The patient initially presented with fever, lethargy, and a bulging anterior fontanelle. Cerebrospinal fluid (CSF) analysis was highly suggestive of bacterial meningitis. However, both CSF culture and the FilmArray ME Panel were negative, likely due to prior empirical antibiotic therapy at another hospital. The causative organism was isolated on blood cultures. The infant was treated with intravenous ceftriaxone for 24 days and discharged in excellent clinical condition. Twelve days later, she was readmitted with a relapse of meningitis and bacteremia caused by the same pathogen. She received combination therapy with cefotaxime and cotrimoxazole for 37 days, followed by cefotaxime monotherapy for another 26 days. Immunological evaluation, including assessment of immunoglobulin levels, lymphocyte subsets, and interferon‐γ receptor and Interleukin‐12 receptor expression, was normal. The patient had a favorable outcome with complete clinical and laboratory recovery and a normal neurodevelopmental outcome at follow‐up. *Salmonella* meningitis is a rare but life‐threatening condition in infants, which may be overlooked when relying on molecular diagnostic panels that do not include this pathogen. Negative CSF PCR results should be interpreted with caution in the presence of typical cytological and biochemical findings of bacterial meningitis. Prolonged antimicrobial therapy of at least 4–6 weeks is essential to prevent relapse and ensure optimal microbiologic and clinical outcomes.

## 1. Introduction

The precise diagnosis of bacterial meningitis is essential, especially in very young febrile infants. Conventional methods for its diagnosis are insensitive and take several days to give a result [1]. Although the cytological and biochemical examination of cerebrospinal fluid (CSF) is useful and readily available, it is nonspecific, since it cannot reliably distinguish between bacterial and viral meningitis [2]. Moreover, with the availability of 3rd‐generation cephalosporins that exhibit excellent central nervous system (CNS) penetration, CSF cultures can be negative in empirically treated children with true bacterial meningitis, despite presenting with typical CSF cytological and biochemical findings, including neutrophilic pleocytosis, increased CSF protein and lactate, and hypoglycorrhachia [3].

FilmArray meningitis/encephalitis (ME) panel is a polymerase chain reaction (PCR) multiplex test designed for the concurrent and rapid identification of 14 pathogens, including seven viruses (cytomegalovirus, enterovirus, Herpes Simplex Virus 1 and 2, human Herpesvirus 6, human parechovirus, and varicella zoster virus), six bacteria (*E. coli* K1, *H. influenzae*, *L. monocytogenes*, *N. meningitidis*, *S. agalactiae*, and *S. pneumoniae*), and one fungus (*Cryptococcus)*. Despite its proven efficacy in identifying the responsible pathogen in case of a negative CSF culture, caution is advised for a negative result in young febrile infants with the CSF findings indicative of bacterial meningitis, as rare cases of Gram‐negative meningitis due to *Salmonella* spp. or other Enterobacteriaceae can be missed.

We present a 2‐month‐old infant with *Salmonella. enteritidis* meningitis and concurrent bacteremia, who relapsed 12 days after receiving 24 days of intravenous ceftriaxone and review the relevant literature.

## 2. Case Presentation

A formula‐fed 2.5‐month‐old female infant was admitted to the pediatric clinic of a county hospital due to low‐grade fever for 4 days with accompanying nasal congestion. On admission, a complete blood count (CBC), biochemical tests, a urinalysis, and urine culture were performed, while two blood cultures were obtained, and a lumbar puncture was performed. The CBC showed leukocytes 19,620/μL (neutrophils 19.6%, lymphocytes 64.5%), hemoglobin 10.1 g/dL, platelets 507,000/μL, with C‐reactive protein (CRP) 2.6 mg/dL. The CSF was colorless, with 2 nucleated cells, protein 27.5 mg/dL, and glucose 47.8 mg/dL. No CSF culture was obtained due to the lack of pleocytosis. Toward the end of the 2nd day, and while she was receiving intravenous ampicillin/sulbactam, she developed moderately severe diarrhea. On the 3rd day of hospitalization, the blood cultures that were obtained on admission grew *Salmonella spp*., so ampicillin/sulbactam was discontinued, and she was empirically started on ceftriaxone 100 mg/kg/day. On the evening of the same day, the anterior fontanelle was pulsating. At the same time, her clinical condition deteriorated, spiking a fever to 39.4°C, along with a marked decrease in oral intake. At that point, she was transferred to us for further management.

On admission, she was afebrile and active but had a pulsating anterior fontanelle. A new CBC disclosed leukocytes 29,910/μL, hemoglobin 8.7 g/dL, and platelets 738,000/μL. The serum CRP had skyrocketed to 25.6 mg/dL. A lumbar puncture was performed that produced slightly cloudy CSF with nucleated cells 5600/μL (neutrophils 73% and lymphocytes 27%), erythrocytes 1000/μL, protein 172 mg/dL, and glucose 97 mg/dL (concurrent blood glucose 102 mg/dL). The CSF culture and a multiplex PCR assay of the CSF (BioMérieux FilmArray ME Panel) were negative.

**FIGURE 1:**
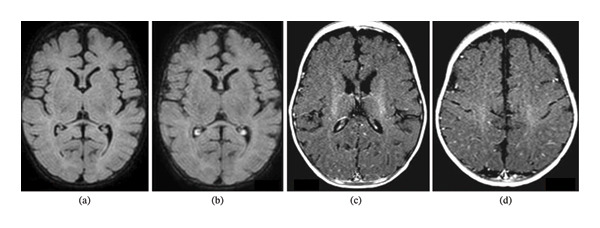
FLAIR sequence pre‐ (a) and post‐Gd (b) failed to attest to higher CSF signal intensity post‐Gd. T1w_Gd demonstrates increased supratentorial leptomeningeal enhancement, most prominently involving occipital regions bilaterally at the level of the superior aspects of the basal ganglia (c), as well as biparietal (d).

*Salmonella* spp. grew on the blood cultures of the referring county hospital, which was identified as *S. enteritidis*, sensitive to ceftriaxone. The complete antibiotic susceptibility testing of the isolated *S. enteritidis* is shown in Table 1. The patient continued treatment with ceftriaxone 120 mg/kg/day in two divided doses. On the 3rd day of hospitalization, in the setting of persistent low‐grade fever, brain MRI was obtained (1.5T, Philips Multiva system, 8 element phased‐array coil). In the protocol used, we implemented, among other sequences, FLAIR pre‐ and post‐Gd to demonstrate potential abnormal CSF signal intensity (Figure 1a,b) as well as T1w pre‐ and post‐Gd to evaluate parenchymal or ventricular involvement. In the context of potential CNS complications, there was no imaging evidence of cerebritis, organized abscess formation, or cerebral empyema within an extra‐axial collection. Furthermore, no abnormal ependymal enhancement suggestive of ventriculitis was identified. However, there was an impression of increased supratentorial leptomeningeal enhancement, most prominently involving occipital regions bilaterally at the level of the superior aspects of the basal ganglia, as well as biparietal (Figure 1c,d). The patient defervesced on the 4th hospital day and remained afebrile and in good clinical condition since then. A follow‐up brain MRI was performed on the 18th hospital day and was unremarkable. On the same day, a new traumatic lumbar puncture showed CSF with 10 leukocytes/μL, protein 65.2 mg/dL, and normal glucose, while the CSF culture remained negative. Two days later, she underwent autoacoustic emissions, which were normal. Eventually, the infant completed 24 days of antibiotic therapy and was sent home in excellent clinical condition. During this period, several stool cultures were obtained, and all yielded negative results for *Salmonella* spp.

**TABLE 1:** Complete antibiotic susceptibility testing for the isolated *Salmonella enteritidis* that was grown in blood culture alone.

Antimicrobial	MIC (mg/L)	Interpretation
Amikacin	2	R
Colistin	≥ 8	R
Amoxicillin/clavulanic acid (other)	≤ 4	S
Amoxicillin/clavulanic acid (urine)	≤ 4	S
Ampicillin	≤ 2	S
Aztreonam	≤ 1	S
Cefotaxime (meningitis)	≤ 0.25	S
Cefotaxime (other)	≤ 0.25	S
Cefoxitin	≤ 4	R
Ceftazidime	≤ 0.12	S
Ceftazidime/avibactam	≤ 0.12	S
Ceftolozane/tazobactam	≤ 0.25	S
Ceftriaxone (meningitis)	≤ 0.25	S
Ceftriaxone (other)	≤ 0.25	S
Cefuroxime axetil	4	R
Ciprofloxacin	0.5	R
Ertapenem	≤ 0.12	S
Fosfomycin	≤ 16	S
Gentamicin	≤ 1	R
Imipenem	≤ 0.25	S
Imipenem/relebactam	≤ 0.25	S
Levofloxacin	0.5	R
Meropenem (meningitis)	≤ 0.25	S
Meropenem (other)	≤ 0.25	S
Meropenem/vaborbactam	≤ 0.5	S
Moxifloxacin	1	R
Piperacillin/tazobactam	≤ 4	S
Tobramycin	≤ 1	R
Cotrimoxazole	≤ 20	S

*Note:* R: resistant; S: susceptible.

Abbreviation: MIC, minimal inhibitory concentration.

Twelve days after hospital discharge, she returned with clinical signs of meningitis, including high‐grade fever, lethargy, and a bulging anterior fontanelle. A repeat lumbar puncture revealed 3460 nucleated cells/μL, protein 205 mg/dL, and marked hypoglycorrhachia, with a CSF glucose of 8 mg/dL. Blood culture once again grew *S. enteritidis*, while the CSF culture remained negative due to the urgent administration of a single dose of ceftriaxone before the lumbar puncture. The new follow‐up brain MRI that was carried out to rule out CNS complications was unremarkable. The patient was started on antibiotic therapy with intravenous cefotaxime (300 mg/kg/day divided every 6 h) and intravenous cotrimoxazole (15 mg/kg/day for trimethoprim divided every 12 h). She became afebrile on the 5th day of hospitalization and remained asymptomatic and in excellent clinical condition thereafter. Cotrimoxazole was discontinued after 37 days due to neutropenia (absolute neutrophil count 730/μL), and the infant continued cefotaxime monotherapy, which she received for a total of 9 weeks. After 60 days of treatment, she underwent an atraumatic lumbar puncture with conscious sedation that showed a completely normal CSF. Table 2 shows the patient’s laboratory workup during hospitalization. At 4‐month follow‐up, the infant remains clinically well and continues to exhibit normal psychomotor development. Laboratory work‐up for possible underlying immunodeficiency that was performed at the age of 6 months showed normal serum immunoglobulins and blood lymphocyte subpopulations, and normal expression of the interferon‐γ (IFN‐γ) and Interleukin‐12 (IL‐12) receptor on leukocytes.

**TABLE 2:** Patient’s laboratory work‐up during hospitalization.

	Day 1 (admission to the county hospital)	Day 5 (admission to us)	Day 18	Day 24	Day 37 (relapse)	Day 1 (after relapse)	Day 5 (after relapse)	Day 37 (after relapse)	Day 57 (after relapse)	Day 60 (after relapse)
WBC (X 10^3^/μL)	19.62	29.91		14.5	23.56	15.34	12.15	8.7	9.93	
NEUT (%)	19.6	62.8		17.5	66	77.4	30.8	8.49 (ANC = 730/μL)	14.7 (ANC = 1450/μL)	
LYM (%)	64.5	30.6		72.6	27	17.1	56	79.8	73	
Hb (g/dL)	10.1	8.7		11.2^∗^	10.1	8.5	7.2^∗^	11	11.7	
Hct (%)	29.7	25.3		33.6	29.1	24.9	22.6	34.2	35.9	
Platelets (X 10^3^/μL)	507	738		573	466	326	271	259	257	
Glucose (mg/dL)	167	102			235	176	85	94	93	92
CRP (mg/dL)	2.6	25.6		< 0.1	3.6	22.1	10.7	< 0.1	< 0.1	
^∗^Patient was transfused										

*CSF*										
Nucleated cells/μL	2	5600 (neut 73% lym 27%)	10 (neut 45% lym 55%)		3460 (neut 80% lym 20%)					5
RBCs		1000	1200		10					0
Protein (g/dL)	27.5	171	65.2		205.7					31.8
Glucose (mg/dL)	47.8	97	63		8					46

^∗^The elevated Hb on that day, compared to previous values, was due to a transfusion of packed red blood cells on that day (day 24).

## 3. Discussion

The genus *Salmonella* is a member of the family Enterobacteriaceae. *Salmonella* spp. are Gram‐negative, non–spore‐forming bacilli that contain three different types of antigens, i.e., somatic O, flagellar H, and capsular Vi antigens. The agglutinating properties of these antigens are used to differentiate among > 2500 serologically distinct types of *Salmonella* [4]. *S. typhimurium* and *S. enteritidis* are the most common serotypes in the United States [5]. Routes of transmission include consumption of contaminated food, such as meat and dairy products, exposure to poultry and swine, contact with reptiles and rodents, such as snakes, turtles, and lizards colonized with the organism, ingestion of contaminated breast milk, exposure to carrier mothers, and, rarely, transplacental transmission [5–9]. The source of *S. enteritidis* infection was never identified in our infant because, despite our suspicion that the formula she consumed was infected, we were unable to find an unopened can with the same lot number to culture its contents. The family resides in a rural area; however, no known contacts with reptiles or rodents were reported. Moreover, not only the patient but also parents and siblings had repeatedly negative stool cultures for *Salmonella*. Although asymptomatic, family members were tested with multiple stool samples over a period of several weeks because of the possibility of intermittent shedding among both convalescent and chronic carriers [10].

The Centers for Disease Control and Prevention estimates that 1.4 million people are affected by nontyphoidal *Salmonella* (NTS) each year in the United States, considering unreported cases [4]. Although *Salmonella* is a frequent cause of community‐acquired gastroenteritis, it only rarely progresses to severe invasive disease such as bacteremia or meningitis [6]. Meningitis caused by *Salmonella* is uncommon, occurring in < 1% of acute bacterial meningitis cases in developed nations. In contrast, in Africa, *Salmonella* is responsible for approximately 13% of meningitis cases, making it the 4th most common bacterial cause, after *S. pneumoniae, H. influenzae, and N. meningitidis* [11]. Self et al. analyzed national surveillance data from 1968 to 2015 for culture‐confirmed *Salmonella* infections among US infants. Among 190,627 reported cases during this period, 86.7% were gastroenteritis, 3.5% were bacteremia, and only 0.2% were meningitis [9].

Acute complications of *Salmonella* meningitis include hydrocephalus, subdural collection, cerebral infarction, ventriculitis, empyema, intracranial abscess, and cranial nerve palsy. Long‐term outcomes among survivors include motor disabilities, developmental delays, epilepsy, hearing loss, microcephaly, visual deficits, speech, and language disorders [12]. Notably, factors contributing to death or serious complications are altered consciousness, a CSF/blood glucose ratio < 0.5, a CSF protein > 200 mg/dL, focal intracranial infection, ventriculitis, cerebral infraction, and seizures during hospitalization [12]. The Glasgow Coma Scale seems to be the best predictor of mortality [13, 14]. Moreover, *Salmonella* spp. meningitis is associated with a high rate of relapse, particularly in young infants. Relapse rates of up to 60% have been reported, with higher rates observed when antimicrobial therapy was administered for < 4 weeks [15, 16]. Relapse usually occurs within days to a few weeks after apparent clinical recovery [17].

The mortality rate of *Salmonella* meningitis ranges from 18% to 58% in children and 89% in neonates in developing countries. Contributing factors include immune deficiency syndromes, malnutrition, and delays in appropriate antibiotic therapy [11]. In a retrospective study from Riyadh, Saudi Arabia, there were 141 patients between 1999 and 2016 aged ≤ 14 years with invasive *Salmonella* infections, of whom 14 had meningitis. Three of those pediatric patients suffered from chronic granulomatous disease (CGD), severe combined immunodeficiency (SCID), and IL‐12 deficiency, respectively [11]. Keddy et al. reviewed the clinical and microbiological characteristics of NTS meningitis in South Africa, where the human immunodeficiency virus (HIV) prevalence is high. They reported that 52.2% of patients aged < 5 years were HIV‐positive [13]. Palyvou et al. suggested that children with *Salmonella* meningitis should be investigated for possible immunodeficiency, as it is a rare infection. Conditions such as Mendelian Susceptibility to Mycobacterial Disease (MSMD), HIV, CGD, sickle cell disease, and leukemia should be considered [18], and further testing, such as evaluation of the IL‐12/IFN‐γ axis, should be performed [18–20]. In our case, the infant was HIV‐negative. Moreover, extensive work‐up for possible underlying immunodeficiency that was performed at the age of 6 months demonstrated normal serum immunoglobulins, blood lymphocyte subpopulations, and expression of the IL‐12 and IFN‐γ Receptor 1 (IFN‐γR1/CD119) on leukocytes.

Analysis of the CSF plays a crucial role in diagnosing meningitis and other CNS infections, but traditional cytological and biochemical tests lack specificity. Microbiological techniques, such as Gram staining, antigen detection, and culture, are often insensitive or require a long time. On the other hand, FilmArray ME Panel can directly detect 14 pathogens from CSF samples using PCR [21]. Obaro et al. compared standard bacterial culture with the FilmArray ME Panel in 400 CSF specimens from patients with suspected meningitis. Of these, 372 were children < 5 years old, and 55 were infants < 2 months. While only 32 cultures were positive, 107 PCR tests yielded positive results. In 3 of 32 cases, the negative result was due to *Salmonella* spp. [22]. A negative FilmArray ME Panel should be interpreted with caution in children, especially young febrile infants, whose CSF findings are suggestive of bacterial meningitis. In rare instances, like ours, Gram‐negative meningitis caused by *Salmonella* spp. or other Enterobacteriaceae may be missed, potentially leading to serious clinical consequences, as meningitis due to these Gram‐negative pathogens typically requires a much longer duration of treatment.

Third‐generation cephalosporins and fluoroquinolones are considered first‐line antibiotics in cases of *Salmonella* meningitis, although chloramphenicol, cotrimoxazole, and ampicillin have also been used either alone or in combination. Price et al. recommended the combination of ceftriaxone and ciprofloxacin for at least 3 weeks after a negative CSF culture. Ciprofloxacin penetrates the CSF well, and the risk of arthropathy appears to be low [23]. The Chinese Medical Association recommends the intravenous antibiotics to be stopped in cases of *Salmonella* meningitis if the child is asymptomatic and afebrile for over a week, the CSF cell count is < 20/μL, the CSF glucose and protein are normal, the CSF culture is negative, and there are no neurological complications [24]. In our case, the patient met all of the above and was initially discharged after 24 days of ceftriaxone monotherapy. However, she relapsed 12 days later, indicating that the duration of the initial treatment was insufficient. Importantly, although serial brain MRI examinations did not reveal intracranial complications, the absence of radiological abnormalities does not preclude completion of the recommended 4–6‐week course of antimicrobial treatment in patients with *Salmonella* meningitis because of the well‐documented risk of relapse. Tissue macrophages phagocytose *Salmonella* spp. and serve as a reservoir in which the bacteria can survive, replicate, and disseminate to the mesenteric lymph nodes, Peyer patches, liver, and spleen. This explains why, despite repeated negative CSF and blood cultures, a relapse can occur if appropriate antibiotic therapy is administered for an inadequate time [25]. Following relapse, we used combination therapy with cefotaxime and cotrimoxazole. In our patient, we did not consider the relapse to be a result of in vivo resistance to ceftriaxone but rather the result of inadequate duration of antibiotic therapy. The decision to switch to cefotaxime was based on its more favorable safety profile in young infants compared with ceftriaxone [26]. The clinical isolate of *S. enteritidis* was resistant to ciprofloxacin, and we did not choose chloramphenicol, as it has been associated with slow responses, frequent complications, and high mortality of up to 60%–80% [16]. We considered using meropenem; however, carbapenems, although effective, have prominent antianaerobic activity that can unfavorably affect the intestinal flora, especially since the treatment of a relapsing infection requires prolonged therapy. On the other hand, cotrimoxazole, albeit being a bacteriostatic drug, demonstrates good antimicrobial activity against many organisms responsible for meningitis, and both of its components reach high concentrations in body tissues, including the CSF. Clinical reports suggest that cotrimoxazole can be useful for treating Gram‐negative bacillary meningitis, particularly when the causative organisms show only partial sensitivity to 3rd‐generation cephalosporins or when they are resistant to these drugs. The efficacy of cotrimoxazole in cases of *Salmonella* meningitis is not unexpected in view of its excellent activity in patients with typhoid fever [27].

While there is no consensus on the optimal duration of treatment for *Salmonella* meningitis, several reports of relapses have been published in patients treated with antibiotics for < 4 weeks [6]. The most recent recommendation of the American Academy of Pediatrics, as stated in the Report of the Committee on Infectious Diseases, is a 4‐week regimen with ceftriaxone [28], while several authors recommend at least 4–6 weeks [6, 11, 29, 30]. At relapse, our patient was treated for 9 weeks and had a favorable outcome, likely due to the administration of two effective antibiotics with excellent CSF penetration, the long duration of therapy, and the prompt hospital presentation at the first sign of relapse. The choice of treatment duration was empirical as no evidence‐based recommendations exist regarding treatment duration after relapse. Considering that our patient had already relapsed following 24 days of ceftriaxone monotherapy, we opted for a more conservative treatment approach to minimize the risk of a second relapse.

## 4. Conclusion

*Salmonella* meningitis represents a rare, life‐threatening infection in infants. Diagnosis may be overlooked, especially following empirical antibiotic administration with 3rd‐generation cephalosporins, since *Salmonella* spp. are not detected in the CSF by commercially available multiplex PCR assays. Relapse may occur despite initially negative cultures, possibly due to the ability of *Salmonella* spp. to survive intracellularly within macrophages and persist despite apparent microbiological clearance. Extended antibiotic therapy is, therefore, essential to reduce the risk of relapse. Finally, even after a thorough diagnostic work‐up, the source of salmonellosis may remain unidentified.

## Author Contributions

Sonia Alexiadou and Elpis Mantadakis contributed to the conception, drafting, and critical revision of the manuscript. Ioanna Irakleidou helped with the search of the medical literature. Stavroula Kalatzi and Eleni Myrtzaki helped with the search of the medical literature and took care of the patient during her hospitalization. Evlampia A. Psatha interpreted the MRI examinations and provided a detailed description of the imaging protocol and findings. Dimitrios Cassimos reviewed, revised, and approved the final submitted version.

## Funding

No specific funding was received for this work.

## Disclosure

All authors have read and approved the final version of the manuscript and agree to be accountable for all aspects of this work.

## Ethics Statement

Our institution does not require ethical approval for reporting individual case reports.

## Consent

Written informed consent was obtained from the mother for her anonymized information to be published in this article.

## Conflicts of Interest

The authors declare no conflicts of interest.

## Data Availability

Data that support the findings of this article are available from the corresponding author.
